# Neuromuscular diseases: genomics-driven advances

**DOI:** 10.1186/s44342-024-00027-y

**Published:** 2024-11-26

**Authors:** Anna Cho

**Affiliations:** 1 Department of Pediatrics, Seoul National University Bundang Hospital, 82, Gumi-ro, 173beon-gil, Bundang-gu, Seongnam-si, Gyeonggi-do 13620 Republic of Korea; 2 Department of Genomic Medicine, Rare Disease Center, Seoul National University Bundang Hospital, 82, Gumi-ro, 173beon-gil, Bundang-gu, Seongnam-si, Gyeonggi-do 13620 Republic of Korea

**Keywords:** Neuromuscular diseases, Genomics, Gene therapy, Next-generation sequencing (NGS), Rare genetic disorders

## Abstract

Neuromuscular diseases (NMDs) are a group of rare disorders characterized by significant genetic and clinical complexity. Advances in genomics have revolutionized both the diagnosis and treatment of NMDs. While fewer than 30 NMDs had known genetic causes before the 1990s, more than 600 have now been identified, largely due to the adoption of next-generation sequencing (NGS) technologies such as whole-exome sequencing (WES) and whole-genome sequencing (WGS). These technologies have enabled more precise and earlier diagnoses, although the genetic complexity of many NMDs continues to pose challenges. Gene therapy has been a transformative breakthrough in the treatment of NMDs. In spinal muscular atrophy (SMA), therapies like nusinersen, onasemnogene abeparvovec, and risdiplam have dramatically improved patient outcomes. Similarly, Duchenne muscular dystrophy (DMD) has seen significant progress, most notably with the FDA approval of delandistrogene moxeparvovec, the first micro-dystrophin gene therapy. Despite these advancements, challenges remain, including the rarity of many NMDs, genetic heterogeneity, and the high costs associated with genomic technologies and therapies. Continued progress in gene therapy, RNA-based therapeutics, and personalized medicine holds promise for further breakthroughs in the management of these debilitating diseases.

## Background

Neuromuscular diseases (NMDs) have experienced significant advancements in diagnosis and treatment, largely driven by breakthroughs in genomics. Before the 1990s, fewer than 30 NMDs had their molecular genetic causes identified [1]. Today, however, over 600 single-gene NMDs have been characterized, with new discoveries continuing to emerge each year [2]. In the past decade, gene therapy research has advanced at an unprecedented rate, leading to the development and approval of therapies for previously untreatable diseases. This progress marks a fundamental shift in the management of NMDs.

## Genetic complexity in neuromuscular diseases

NMDs represent a group of rare disorders with significant clinical diversity and genetic complexity. These diseases are classified into various subgroups, including muscular dystrophies, congenital myopathies, distal myopathies, metabolic myopathies, myotonic syndromes, congenital myasthenic syndromes, motor neuron diseases, hereditary motor and sensory neuropathies, and ion channel muscle diseases [2]. Each subgroup shares multiple causative genes, which means that different molecular genetic diagnoses can be derived from similar clinical symptoms, and the same gene may result in different phenotypes. Since the initial application of next-generation sequencing (NGS) in NMD patients in 2010 [3], this technology has revolutionized the field by enabling the efficient analysis of multiple genes simultaneously. NGS technologies, including whole-exome sequencing (WES) and whole-genome sequencing (WGS), have significantly accelerated the discovery of novel disease-causing genes in NMDs [4, 5]. These technologies have enabled more precise and earlier diagnoses, improving disease management and treatment planning. Targeted panel NGS or WES is now being adopted as a standard in clinical NMD diagnosis, while emerging tools like transcriptome analysis and long-read sequencing further expand the field [6–8].

While NGS has greatly advanced the diagnosis of NMDs, the genetic complexity, particularly the diversity of causal variants, continues to make accurate diagnosis challenging. For numerous major NMDs, molecular diagnosis remains infeasible with NGS. For example, Duchenne muscular dystrophy (DMD) shows genetic heterogeneity, with around 80% of patients having exon deletions or duplications in the dystrophin gene, and about 20% presenting with sequence variants, including intronic mutations in 5%, which complicates diagnosis [9]. In spinal muscular atrophy (SMA), over 95% of patients exhibit homozygous deletion of exon 7 in the *SMN1* gene [10]. Myotonic dystrophy type 1 (DM1) is caused by the expansion of a CTG trinucleotide repeat in the noncoding region of the *DMPK* gene [11], while facioscapulohumeral muscular dystrophy type 1 (FSHD1) is characterized by the contraction of the *D4Z4* repeat array on chromosome 4q35 [12]. Moreover, in metabolic myopathies, mitochondrial DNA mutations are a critical diagnostic factor, often presenting as either single nucleotide variants or large deletions [13]. Additionally, certain genetic disorders, such as Fukuyama congenital muscular dystrophy, show genotypic differences across ethnicities, necessitating tailored diagnostic approaches [14, 15]. These examples underscore the genetic diversity and complexity associated with NMDs, highlighting the challenges of achieving accurate molecular diagnosis even in the era of NGS technologies (Table 1).

**Table 1:** Complexity of diagnostic genetic testing in major neuromuscular diseases

Phenotype	Gene	Molecular genetic testing methods	Proportion of probands with a pathogenic variant detectable		
Duchenne/Becker muscular dystrophy (DMD/BMD)	*DMD*	Sequence analysis	20–35%		
		Gene-targeted deletion/duplication analysis	65–80%		
Spinal muscular atrophy (SMA)	*SMN1*	Sequence analysis	2–5%		
		Gene-targeted deletion/duplication analysis	95–98%		
Facioscapulohumeral muscular dystrophy (FSHD)	*D4Z4*	Targeted analysis for pathogenic variants (pathogenic contraction of number of *D4Z4* repeats)	~ 95%		
		Methylation analysis	~ 5%		
Myotonic dystrophy type 1 (DM1)	*DMPK*	Targeted analysis for pathogenic variants (testing to quantitate the number of *DMPK* CTG trinucleotide repeats)	100%		
Fukuyama congenital muscular dystrophy	*FKTN*	Targeted analysis	Japanese	Non-Japanese Asian	Non-Asian
		c.*4392_*4393insAB185332.1	98% 3	77% 4, 5	0%
		c.647 + 2084G > A	8%	38% (Korean)	0%
		c.139C > T	7%	60% (Chinese)	Rare

## Gene therapy: a new era in NMD treatment

Advancements in genetics have also led to significant progress in the treatment of NMDs, with one of the most notable achievements being the development of gene therapies, which have substantially impacted the therapeutic landscape of these disorders. SMA is a prime example, with the antisense oligonucleotide (ASO) therapy nusinersen (Spinraza) nearing its tenth year since its successful introduction [16, 17]. Following this, the development and clinical application of onasemnogene abeparvovec (Zolgensma), an adeno-associated virus (AAV)-based gene therapy [18, 19], and risdiplam (Evrysdi), a small molecule oral drug [20, 21], have ushered in a new era of treatment options for SMA. All three SMA therapies have demonstrated significant efficacy in altering the natural course of SMA, notably by prolonging ventilation-free survival and enabling the achievement of major motor milestones [16–21]. These treatments achieve optimal outcomes when administered at the earliest possible stage, ideally prior to symptom onset. Accordingly, we are now in an era emphasizing newborn screening [22], with the USA implementing SMA screening for all newborns as of January 2024. Similarly, in DMD, exon-skipping therapies [23, 24] marked a major advancement, and more recently, the FDA approval of delandistrogene moxeparvovec (Elevidys), the first micro-dystrophin gene therapy [25], represents another significant milestone (Table 2). With more gene therapies under development and clinical trials actively ongoing, the future of NMD treatment holds great promise for further breakthroughs.

**Table 2:** Dystrophin restoration drugs approved and in clinical development

Name	Company	Therapeutic strategies	Approval or clinical stage
Eteplirsen	Sarepta Therapeutics	Exon 51 skipping	FDA
Viltolarsen	NS Pharma	Exon 53 skipping	FDA
Golodirsen	Sarepta Therapeutics	Exon 53 skipping	FDA, JAPAN
Casimersen	Sarepta Therapeutics	Exon 45 skipping	FDA
Delandistrogene moxeparvovec (SRP-9001)	Sarepta Therapeutics	Micro-dystrophin	FDA
PF-06939926	Pfizer	Micro-dystrophin	Phase III
SGT-001	Solid Biosciences	Micro-dystrophin	Phase I/II
RGX-202	REGENXBIO	Micro-dystrophin	Phase I/II

## Challenges and future directions

Despite the progress in the field, there remain several challenges and limitations. One of the main obstacles is the clinical rarity of many NMDs, making large-scale clinical trials difficult to conduct. The heterogeneity in disease presentation and progression further complicates the development of universal treatments. Moreover, the genetic complexity of NMDs, with its diverse range of mutations and variable phenotypes, presents challenges in both diagnosis and treatment. In many cases, the specific mutation causing the disease may still be unknown, and even when the mutation is identified, developing effective therapies can be difficult due to the multifaceted nature of the underlying pathophysiology. Another major limitation is the cost and accessibility of advanced genomic technologies and therapies. Gene therapies, while promising, are often expensive and not readily available in all regions, limiting their impact on the broader patient population. Ensuring equitable access to these treatments remains a critical challenge for the future. The future of neuromuscular disease research and treatment lies in continued advancements in gene therapy and RNA-based therapeutics.

## Conclusions

Neuromuscular diseases represent a group of rare yet complex disorders that have benefited significantly from advancements in genomics. The identification of over 600 disease-causing genes, coupled with the development of gene therapies, has brought hope to patients suffering from previously untreatable conditions. However, challenges such as genetic heterogeneity, clinical rarity, and accessibility to treatments remain. The future of NMD research promises continued innovation, with gene editing, RNA-based therapies, and personalized medicine leading the way towards more effective and equitable treatments for these debilitating diseases.

## Data Availability

No datasets were generated or analysed during the current study.

## Abbreviations

AAV: Adeno-associated virus
ASO: Antisense oligonucleotide
DMD: Duchenne muscular dystrophy
NGS: Next-generation sequencing
NMD: Neuromuscular disease
SMA: Spinal muscular atrophy
WES: Whole-exome sequencing
WGS: Whole-genome sequencing
